# Association between DNA methylation and multidrug resistance in human glioma SHG-44 cells

**DOI:** 10.3892/mmr.2014.2690

**Published:** 2014-10-17

**Authors:** JIN CHEN, ZHONG-YE XU, FENG WANG

**Affiliations:** Department of Neurosurgery, The Second Affiliated Hospital of Chongqing Medical University, Chongqing 400010, P.R. China

**Keywords:** DNA methylation, multidrug resistance, gliomas, SGH-44, adriamycin

## Abstract

The aim of the present study was to evaluate the association between DNA methylation and multidrug resistance (MDR) in glioma and identify novel effectors responsible for MDR in human gliomas. An MDR glioma cell line, SGH-44/ADM, was developed using adriamycin (ADM) impulse treatment. Cryopreservation, recovery and withdrawal were performed to evaluate the stability of SGH-44/ADM cells. The adherence rate and cellular morphology were observed by microscopy, and the cell growth curve and doubling time were determined. DNA methylation was analyzed using a methylated DNA immunoprecipitation microarray chip (MeDIP-Chip). The cell cycle, Rh123 ingestion and exudation, and SGH-44/ADM apoptosis were analyzed by flow cytometry. SGH-44/ADM cells showed little difference as compared with parental cells, except that SGH-44/ADM cells were bigger in size with a wizened nucleus. Compared to SGH-44 cells, a larger proportion of SGH-44/ADM cells remained in G1 and S phase, as measured by flow cytometry. The MDR of SGH-44/ADM was associated with the upregulation of multi-drug resistance 1, prostaglandin-endoperoxide synthase 2 (COX-2); protein kinase C α (PKCα); however, the expression of these genes was not associated with DNA methylation. In the MeDIP-Chip analysis, 74 functions were markedly enhanced, and seven significant pathways were observed. Genes including *SNAP47*, *ARRB2*, *PARD6B*, *TGFB1*, *VPS4B* and *CBLB* were identified by gene ontology analysis. The predominant molecular mechanism of MDR in SGH-44/ADM cells was identified as exocytosis and efflux. The expression of COX-2, PKCα and P-glycoprotein (Pgp) was not found to be associated with DNA methylation. Genes including *SNAP47*, *VAMP4* and *VAMP3* may serve as the downstream effectors of Pgp, COX-2 or PKCα; however, further experiments are required to verify these observations.

## Introduction

A glioma is a tumor that originates in the brain or spine, arising from glial cells. The most common site of gliomas is in the brain (1). Gliomas attribute for ~30% of all brain and central nervous system tumors, and ~80% of all malignant brain tumors. The anthracyclines, such as adriamycin (ADM), are among the most effective anticancer treatments, and are effective against more types of cancer than any other class of chemotherapeutic agents (2–4). However, the effect of chemotherapy on the survival rate in patients with malignant glioma is low, giving an improvement in survival rate of 6% after one year and 5% after two years (5). This poor response to therapy may be due to multi-drug resistance (MDR) (6).

Mechanisms to explain drug resistance have been reported to include the blood-brain barrier (BBB), efflux pumps, DNA repair and cancer stem-like cells (7–9). P-glycoprotein (Pgp), the *MDR1* gene product in humans, is a drug efflux pump which has a significant role in modulating MDR in a wide variety of human cancers (10,11). Pgp is highly expressed in the BBB, with weak expression in tumor cells (12). MDR-associated protein 1 (MRP1), expressed in glioma cell lines, has been suggested to be involved in MDR both *in vivo* and *in vitro* (13,14). Low density lipoprotein receptor-related protein (LRP) is a member of the low density lipoprotein (LDL) receptor (15). A previous study showed that the association of statins, plus an LDL receptor-targeted liposomal drug, may increase *in vitro* drug delivery across the BBB (16). It has been previously observed that the expression levels of prostaglandin-endoperoxide synthase 2 (COX-2), protein kinase C (PKC) and Activator Protein 1 were significantly upregulated in drug resistant cell lines that overexpress MDR1/Pgp170, suggesting that there is a potential link between COX-2, PKC and MDR1/Pgp170 (17). Glutathione S transferase π (GSTπ) is another protein that catalyzes conjugation reactions of glutathione (GSH), linking to cytotoxic drugs and leading to their inactivation (18). In addition, the Fanconi anemia (FA) pathway is involved in glioma drug resistance (19).

The pattern of methylation is an important line of research in MDR (20–22). Recent research has demonstrated that acquired gemcitabine resistance is associated with DNA promoter methylation-independent human equilibrative nucleoside transporter 1 and deoxycytidine kinase gene downregulation and hyper-expression of G9A methyltransferase (21). The death-associated protein kinase (DAPK) is hypermethylated in drug-resistant derivatives generated from both non-small cell lung cancer (NSCLC) and head and neck squamous cell carcinoma (HNSCC) cell lines (22). Expression of O6-methylguanine-DNA methyltransferase is associated with resistance to radiotherapy in gliomas (23). However, the association between methylation and MDR in glioma has yet to be evaluated.

In the present study, a stable MDR glioma cell line, SGH-44/ADM, was generated and the association between DNA methylation and MDR was analyzed using a methylation DNA immunoprecipitation microarray chip (MeDIP-Chip). The findings from this study may provide novel insight for the treatment of gliomas.

## Materials and methods

### Cell culture and construction of an ADM resistant SGH-44/ADM cell line

The human glioma cell line SHG-44 (Medicine College of Suzhou University, Suzhou, China) was routinely cultured in RPMI 1640 medium (Gibco-BRL, Invitrogen Life Technologies, Carlsbad, CA, USA) supplemented with 5 or 10% heat-inactivated fetal bovine serum (FBS; HyClone Laboratories, Inc., Logan, Utah, USA), l00 U/ml penicillin and 100 U/ml streptomycin (Qilu Pharmaceutical Co., Ltd., Jinan, China). An ADM-resistant cell line of SHG-44, named as SHG-44/ADM, was obtained by culturing the cells in the presence of gradually increasing doses of ADM (Shenzhen Main Luck Pharmaceuticals Inc., Shenzhen, China). In brief, SHG-44 cells were cultured in RPMI 1640 medium with 0.01 mg/ml ADM. The medium was removed and replaced after 48 h, and the cells were cultured to resume normal growth. The cells were then transferred and treated with 0.1 mg/ml ADM. The above process was repeated to obtain ADM-resistant cells. After eight months, cells cultured in 0.1 μM ADM were harvested as the ADM-resistant cell line.

### Antitumor drug resistance of SHG-44 and SGH-44/ADM

The proliferation inhibition of simvastatin on glioma cell lines was determined using a WST-8 Cell Counting Kit-8 (CCK-8; Beyotime Institute of Biotechnology, Haimen, China). The cells were seeded in fresh RPMI 1640 medium containing 10% fetal calf serum 24 h prior to the experiments. Cells were seeded at an initial concentration of 1×10^5^ cells/ml in 100 μl medium in 96-well culture plates with various concentrations of ADM, epinephrine (EPI; Harvest Pharmaceutical Co., Ltd., Shanghai, China), vincristine (VCR; Shenzhen Main Luck Pharmaceuticals Inc.), mitomycin C (MMC; Nanjing Duoyan Biochemistry Co., Ltd., Nanjing, China), arabinofuranosyl cytidine (Ara-C; Nanjing Duoyan Biochemistry Co., Ltd.,) and cisplatin (DDP; Qilu Pharmaceutical Co., Ltd.) for 72 h. Then 20 μl of CCK-8 staining solution was added to each well for 2 h at 37°C before the end of the incubation. The absorbance at 450 nm was measured using a microplate reader (Model 550; Bio-Rad Laboratories, Inc., Hercules, CA, USA). The half inhibitory concentration (IC_50_) and resistance index (RI) were calculated.

### Effect of cryopreservation, recovery and withdrawal on the RI

SHG44/ADM cells were stored in liquid nitrogen, recovered after three months, and then the RI was measured. In addition, the RI of SHG44/ADM cells, which were cultured without ADM for one month, was also measured.

### Analysis of adherence rate, cellular morphology, cell growth curve and doubling time

The cellular morphology was observed using an inverted microscope (IX-71, Olympus Corporation, Tokyo, Japan). Cells were seeded at an initial concentration of 1×10^5^ cells/ml in 24-well culture plates. The pelagic cells were counted every two hours in an eight-hour period. The adherence rate (AR) was calculated as (1 - C_ad_/C_total_) × 100%. Cells were seeded at an initial concentration of 5×10^5^cells/ml in 24-well culture plates, and the viable cells were counted every day for one week. The cell growth curve was constructed, and the doubling time was calculated using the Patterson formula: Td = T × lg2/(1gN2-lgN1). Where Td is doubling time; T is time interval; N2 is end point cell number; and N1 is initial cell number.

### Double layer soft agar detection for colony formation

Cells were diluted to 125, 250 and 500 cells/ml with culture medium, respectively. The diluted cells were then mixed 1:1 with 0.7% agar and poured on 1.2% concretionary agar. When the upper layer was curdled, the cells were cultured for seven days at 37°C with 5% CO_2_. The colony with >50 cells was counted.

### Flow cytometry for cell cycle analysis

The measurement of the cell cycle was performed by flow cytometry (BD FACSCalibur™, BD Biosciences, Franklin Lakes, NJ, USA). After synchronization, the cells were cultured in fresh medium with 10% FBS to 90% confluency, harvested and suspended with 0.3 ml phosphate buffered saline (PBS) containing 5% FBS. A volume of 0.7 ml ethanol was added for immobilization. The cells were stored at −20°C for 24 h and washed with PBS. RNase A, 0.1 mg, was added for RNA degradation, then the cells were placed in the dark for 10 min after adding 0.2 μg propidium iodide (PI). The percentages of cells in different phases were detected by flow cytometry.

### Methylated DNA immunoprecipitation microarray chip (MeDIP-Chip) and analysis

The DNA of SHG-44 and SHG-44/ADM was extracted using a DNA extraction kit (Qiagen, Hilden, Germany). The methylated DNA was purified using EpiQuik Methylated DNA Immunoprecipitation Kit (Qianchen Biotechnology Company, Shanghai, China). The target DNA was amplified with primer A and digested with *Udp* and *Ape*I restriction enzymes. The DNA was mixed with 12 μl TdT buffer, 2 μl TdT and 1 μl DNA Labeling reagent. The mixture was used for gel shift analysis. The data analysis was performed as previously described (24).

### Pathway analysis of MeDIP-Chip

Pathway analysis was performed according to the Kyoto Encyclopedia of Genes and Genomes (KEGG; www.genome.jp/kegg/), BioCarta (www.biocarta.com) and Reactome (www.reactome.org). In addition, Fisher’s exact test and χ^2^ test was performed to select the significant pathways, and the threshold of significance was defined by P-value and false discovery rate (FDR). The enrichment (Re) was calculated as Re = (n_f_/n)/(N_f_/N), where n_f_ was the number of flagged genes within the particular category, n was the total number of genes within the same category, N_f_ was the number of flagged genes in the entire microarray and N was the total number of genes in the microarray (25–27).

### Gene ontology (GO) analysis of MeDIP-Chip

GO analysis was applied to analyze the main functions of the differentially expressed genes according to the GO, which was the key functional classification of the National Center for Biotechnology Information (NCBI), which can organize genes into hierarchical categories and uncover the gene regulatory network on the basis of biological processes and molecular function (28,29). Specifically, a two-tailed Fisher’s exact test and χ^2^ test was used to classify the GO category, and the FDR (30) was calculated to correct the P-value. The smaller the FDR, the smaller the error in judging the P-value. The FDR was defined as *FDR* = 1 - *N_k_*/*T*, where *N_k_* referred to the number of Fisher’s test P-values < χ^2^ test P-values. The P-values were computed for the GOs of all the differential genes. Enrichment provided a measure of the significance of the function. As the enrichment increased, the corresponding function was more specific. This helped identify those GOs with a conclusive function description in the experiment. Within the significant category, the enrichment Re was given by: Re = (*n_f_*/*n*)/(*N_f_*/*N*) with the variables defined as stated above (31).

### Expression analysis of MDR1, MRP1, LRP1, PKCα and COX-2

Total RNA of the lymph node was isolated using TRIzol™ reagent (Invitrogen Life Technologies). The reverse transcription reaction was conducted using random primers and the SuperScript III first-strand synthesis system (Invitrogen Life Technologies). The quantitative polymerase chain reaction (qPCR) was conducted using the following conditions: 95°C for 30 sec followed by 40 cycles of 94°C for 5 sec and 60°C for 30 sec, and dissociation curve analysis of the amplification products was performed at the end of each PCR reaction to confirm that only one product was amplified and detected. Each sample was run in triplicate together with the internal control gene, *GAPDH*. Data analysis of the qPCR was performed with Rotor Gene 6000 Series Software (Corbett Life Sciences, Sydney, Australia). The specific primer sequences (Invitrogen, Shnaghai, China) are listed in Table I.

The SGH-44/ADM and SGH-44 cell lines were washed three times with PBS and cell lysates were prepared in lysis buffer. Protein concentration was estimated using the bicinchoninic acid assay (Pierce Biotechnology, Inc., Rockford, IL, USA) and 20 mg of protein was loaded in each lane. The proteins were resolved by 10% SDS-PAGE. The gel was transferred to a nitrocellulose membrane and blocked with 5% non-fat dry milk (NFDM) for one hour at room temperature. The blot was then incubated with primary antibodies for overnight at 4°C in 2.5% NFDM. Blots were washed with Tris-buffered saline containing 0.1% Tween 20 three times and subsequently incubated with horseradish peroxidase-conjugated goat-anti-rabbit immunoglobulin G antibody (Pierce Biotechnology, Inc.). Enhanced Chemiluminescence substrate (Pierce Biotechnology, Inc.) was used to detect the signal. The blots were stripped and re-probed with mouse monoclonal β-actin antibody, which served as an internal control. All the antibodies were purchased from Cell Signaling Technology, Inc. (Danvers, MA, USA).

### Rhodamine-123 (Rh123) ingestion and exuding of SGH-44/ADM

The SGH-44 and SGH-44/ADM cells were diluted to 1×10^6^ cells/ml with medium. Rh123 (Sigma-Aldrich) was added to the cells to a final concentration of 1 μg/ml. The cells were incubated at 37°C for 1 h and washed three times with PBS. The relative fluorescence intensity of Rh123 accumulated in the cells was measured by flow cytometry at 4°C. To detect the Rh123 exudation, the cells were diluted with RPMI 1640 medium and incubated for 2 h. Then the cells were washed three times with PBS and the relative fluorescence activity of Rh123 was measured again.

### Apoptosis of SGH-44 and SGH-44/ADM

SGH-44 and SGH-44/ADM cells were collected and diluted in PBS. Annexin V-fluorescein isothiocyanate (FITC; Nanjing KeyGen Biotech. Co., Ltd., Nanjing, China), 195 μl, was added to 5×10^4^ cells and the solution (1 μg/ml) was incubated in the dark for 10 min and centrifuged. Following this, 190 μl Annexin V-FITC and 10 μl propidium iodide (PI; Molecular Probes^®^ Life Technologies, Carlsbad, CA, USA) was added. The solution was analyzed by flow cytometry. To evaluate the effect of MDR1 on the MDR of SGH-44/ADM, cyclosporin A (CsA; Sandoz, Holzkirchen, Germany), an MDR1 inhibitor, was added to the SGH-44/ADM cells. A fluorometric assay was performed as previously described (32).

## Results

### Antitumor drug sensitivities and stability of SGH-44/ADM

The IC_50_ to ADM of wild-type SGH-44 was 1.67 μg/ml, while the IC_50_ to ADM of SGH-44/ADM was 17.83 μg/ml. The RI of SGH-44/ADM was 10.7, and following freezing and recovery of the cells, the RI was 10.5. If the ADM was withdrawn, the RI of SGH-44/ADM was reduced to 9.3. The resistance of SGH-44/ADM to other antitumor drugs is shown in Table II.

### Adherence rate, cellular morphology, cell growth curve and doubling time

**Figure 1:**
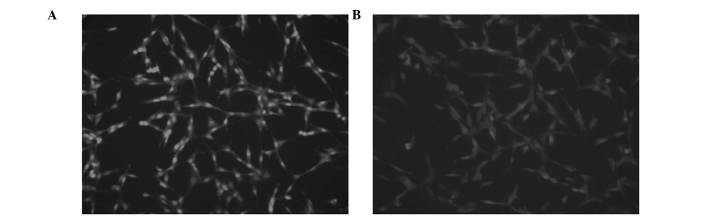
Cellular morphology of SHG-44 and SHG-44/ADM cells (magnification, ×100). (A) SHG-44 and (B) SHG-44/ADM cells. SHG-44/ADM, human glioma cell line SHG-44 with adriamycin resistance.

The cell shape of SGH-44 and SGH-44/ADM cells is shown in Fig. 1. In the drug resistance formation process, the SGH-44 cells were shaped as elongated fibers, uniform in size, tightly packed, adherent to the plate and grown with a clear boundary. Upon administration of the drugs, the surviving cells were not uniform in size, weakly adherent to the plate and shaped with a clear boundary. The most significant difference between the SGH-44 and SGH-44/ADM cells was that the SGH-44 cells had larger nuclei as compared with SGH-44/ADM cells. With extension of the incubation time and number of passages, this emergent abnormal state was reduced. Finally, SGH-44/ADM cells showed minimal differences as compared with parental cells, apart from SGH-44/ADM cells being bigger in size, and with a wizened nucleus.

**Figure 2:**
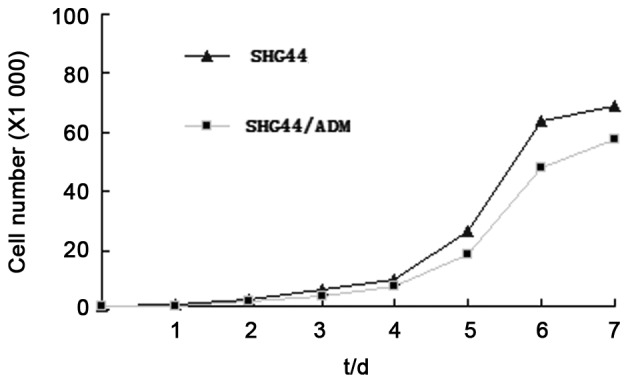
Cell growth curve of SHG-44 and SHG-44/ADM cells. t/d, generation time; SHG-44/ADM, human glioma cell line SHG-44 with ADM resistance; ADM, adriamycin.

The adherence rate was calculated in order to evaluate the viability of SGH-44/ADM cells. The adherence rate of SGH-44 cells at 0, 2, 4, 6 and 8 h was 0, 68.5, 86.6, 93.5 and 96.8%, respectively. The adherence rate of SGH-44/ADM cells at 0, 2, 4, 6 and 8 h was 0, 62.3, 81.5, 91.6 and 94.7%, respectively. The growth curve is shown in Fig. 2. SGH-44/ADM cells showed a slower growth rate as compared with parental cells. The doubling time of SGH-44/ADM was 32.22 h as compared with 26.67 h for SGH-44 cells.

### Improvement of the rate of colony formation in SGH-44/ADM cells

The colony formation rate was calculated as the percentage of cells which could form small groups when dispersed into single cells. Double-layer soft agar experiments showed that the colony formation rate of SGH-44 cells was 20%, whereas the colony formation rate of SGH-44/ADM cells was significantly improved to 52% (P<0.01).

### Analysis of the cell cycle by flow cytometry

**Figure 3:**
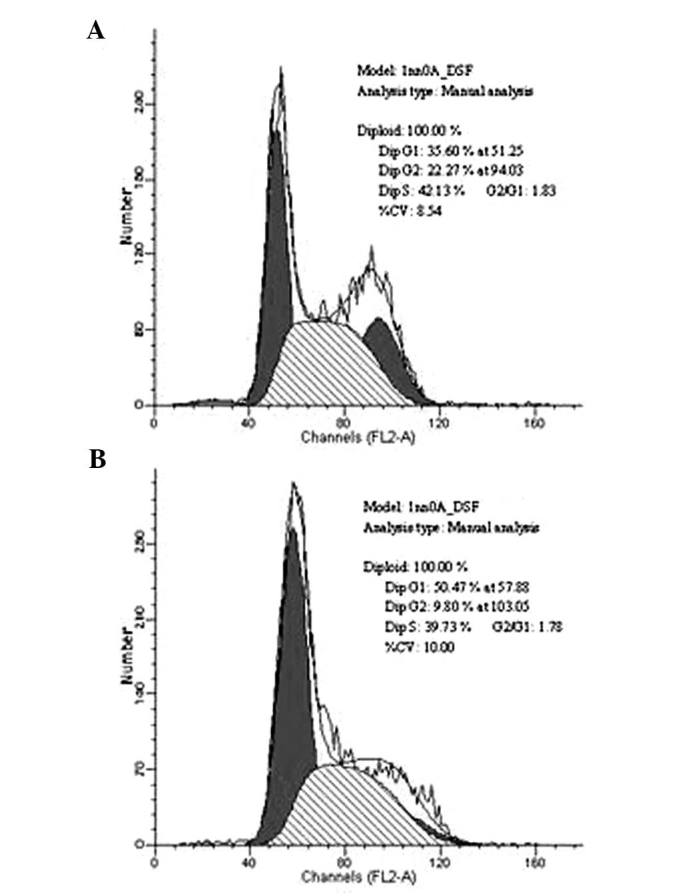
Cell cycle analysis of SGH-44 and SGH-44/ADM cells, measured by flow cytometry. (A) SHG-44 and (B) SHG44/ADM cells. SHG-44/ADM, human glioma cell line SHG-44 with adriamycin resistance.

Flow cytometry was used to evaluate the percentage of SGH-44 and SGH-44/ADM cells in different phases of the cell cycle. The percentage of cells in G1 phase was increased from 35.6 to 50.4% when SGH-44 acquired ADM resistance. The percentage of cells in S phase was 39.7% in SGH-44/ADM cells (Fig. 3).

### GO analysis by MeDIP-Chip

**Figure 4:**
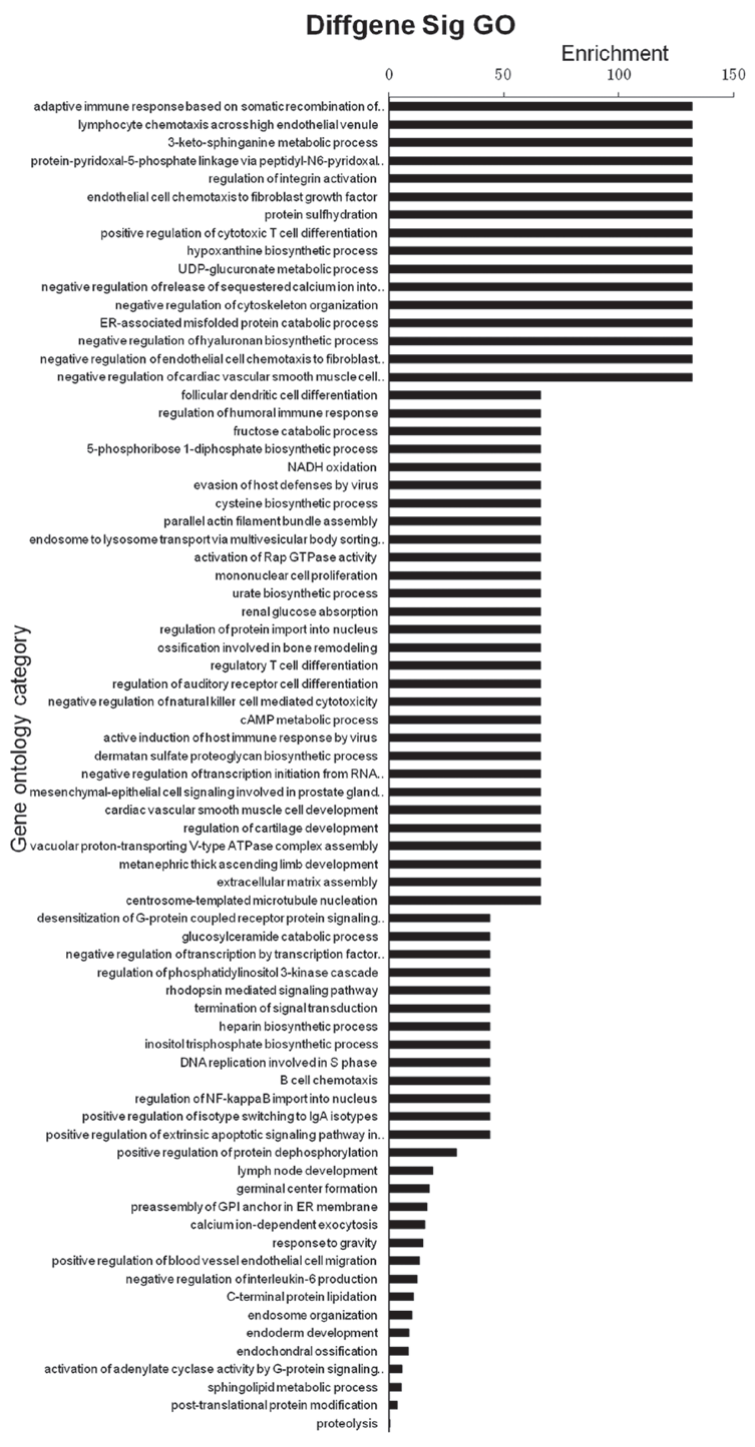
Functional enrichment of the differentially expressed genes, identified between SGH-44/ADM and SGH-44 cells (P<0.05). Sig GO, significant gene ontology; SHG-44/ADM, human glioma cell line SHG-44 with adriamycin resistance.

A functional enrichment analysis was performed using the Web-based Gene Set Analysis Toolkit (33,34) and 74 significantly enhanced functions were identified (Fig. 4; FDR < 0.05). Immune-associated reactions, including the adaptive immune response based on somatic recombination of immune receptors built from immunoglobulin superfamily domains, lymphocyte chemotaxis across the high endothelial venule and positive regulation of cytotoxic T-cell differentiation were significantly enriched (enrichment = 131; P<0.05).

Metabolic processes, including the 3-keto-sphinganine metabolic, hypoxanthine biosynthetic, UDP-glucuronate metabolic, endoplasmic reticulum-associated misfolded protein catabolic and negative regulation of hyaluronan biosynthetic processes were enriched (enrichment = 131; P<0.05).

### Pathway enrichment analysis by MeDIP-Chip

**Figure 5:**
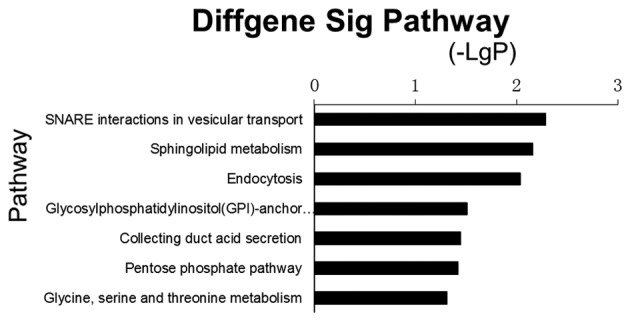
Pathway enrichment in MeDIP-Chip.

Pathway enrichment analysis of all genes involved in MeDIP-Chip was performed using the KEGG automatic annotation server. A total of seven significant pathways with P<0.05 were enriched (Fig. 5). The most significant pathway was synaptosomal-associated protein (SNAP) receptor (SNARE) interaction in vesicular transport (path_id=4130), where P=0.005. Three genes were involved in this pathway, including *SNAP47*, vesicle associated membrane protein (*VAMP*)*4* and *VAMP3*. The other significantly enriched pathways included sphingolipid metabolism, endocytosis, glycosylphosphatidylinositol (GPI)-anchor biosynthesis, collecting duct acid secretion, pentose phosphate pathway and glycine, serine and threonine metabolism. Genes associated with endocytosis included charged multivesicular body protein 1b (*CHMP1B*), arrestin β2 (*ARRB2*), par-6 family cell polarity regulator β (*PARD6B)*, transforming growth factor β1 (*TGFB1*), vacuolar protein sorting 4 homolog B (*VPS4B*) and cbl proto-oncogene, E3 ubiquitin protein ligase B (*CBLB*).

### mRNA and protein expression levels of MDR1, MRP1 and LRP

**Figure 6:**
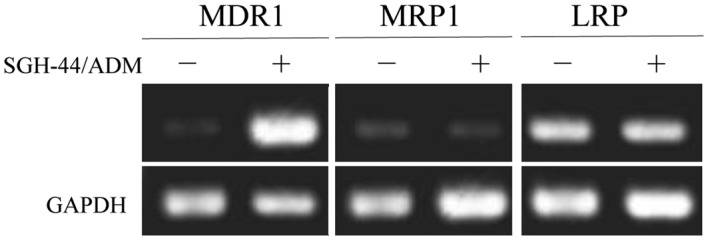
MDR1, MRP1 and LRP mRNA expression in SHG-44/ADM cells. MDR1, multidrug resistance 1; MRP1, MDR-associated protein 1; LRP, low density lipoprotein receptor-related protein 1. SHG-44/ADM, human glioma cell line SHG-44 with adriamycin resistance.

**Figure 7:**
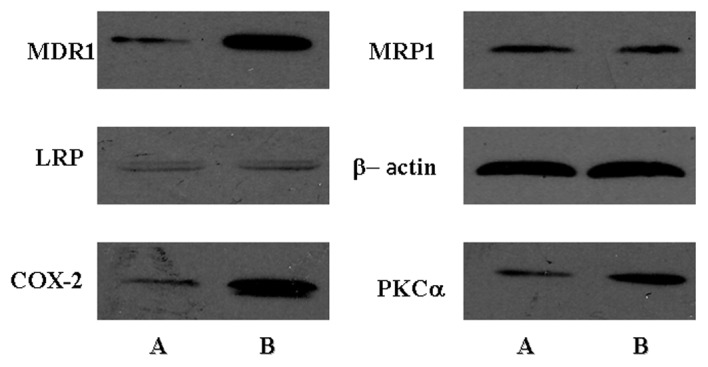
Protein expression of MDR1, MRP1, LRP, COX-2 and PKCα. (A) SHG-44 and (B) SHG44/ADM cells. MDR1, multidrug resistance 1; MRP1, MDR-associated protein 1; LRP, low density lipoprotein receptor-related protein 1; COX-2, prostaglandin-endoperoxide synthase 2; PKCα, protein kinase C α.

The expression of *MDR1*, *MRP1* and *LRP* at the mRNA levels are shown in Fig. 6. The expression of *MDR1* at the mRNA level were significantly increased in SGH-44/ADM cells as compared with SGH-44 cells. The expression of MRP1 and LRP remained unchanged in the two cell lines at the mRNA and protein level. The expression of MDR1 at the protein level was increased in SGH-44/ADM cells as compared with SGH-44 cells, which was consistent with the mRNA expression levels. In addition, the expression of COX-2 and PKCα was increased in SGH-44/ADM cells. The protein expression levels are shown in Fig. 7.

### Rh123 ingestion and exudation in SGH-44/ADM cells

**Figure 8:**
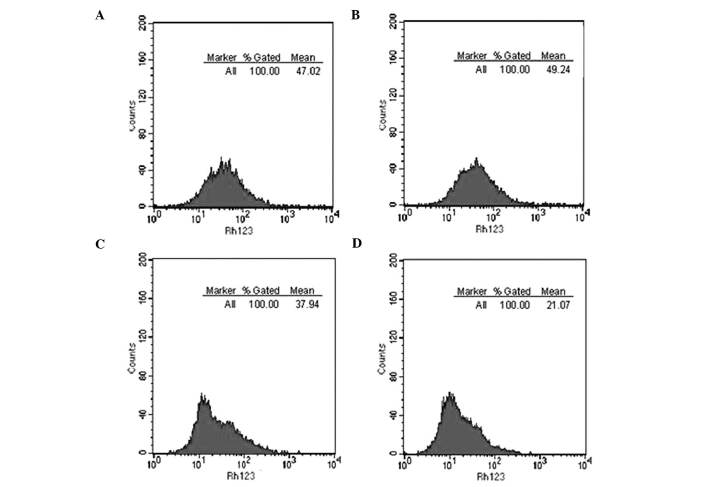
Rh123 accumulation in SGH-44 and SGH-44/ADM cells. (A) SHG-44 and (B) SHG-44/ADM cells treated with 1 μg/ml Rh123 for 1 h. (C) SHG-44 and (D) SHG-44/ADM cells treated with 1 μg/ml Rh123 for 1 h and then cultured without Rh123 for 2 h.Rh123, rhodamine-123; SHG-44/ADM, human glioma cell line SHG-44 with adriamycin resistance.

An Rh123 ingestion and exudation experiment was performed by flow cytometry (Fig. 8). The amount of Rh123 intake by SHG-44 and SHG-44/ADM cells was 43.76±3.65 and 45.87±3.36 (relative fluorescence intensity), respectively. Following effusion, the residual amount of Rh123 in SGH-44 and SGH-44/ADM cells was 35.43±2.17 and 18.49±3.54, respectively. SHG44/ADM cells showed no difference in Rh123 ingestion as compared with SGH-44 cells, but significantly exuded more Rh123 than SGH-44 cells (P<0.01).

### Analysis of apoptosis in SGH-44/ADM cells by flow cytometry and fluorescence microscopy

**Figure 9:**
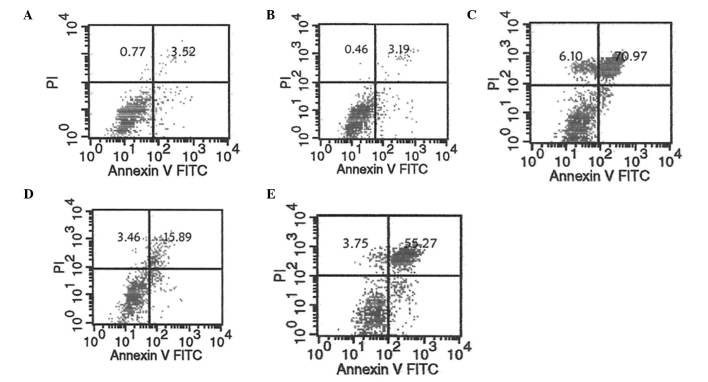
Cell apoptosis rate determined by flow cytometry. (A) SHG-44 and (B) SHG44/ADM cells without any treatment. (C) SHG-44 and (D) SHG44/ADM cells treated with 10 μg/m1 ADM for 48 h. (E) SHG44/ADM cells treated with 10 μg/m1 ADM and 100 μM cyclosporin A for 48 h. ADM, adriamycin; FITC, fluorescein isothiocyanate; PI, propidium iodide; SHG-44/ADM, human glioma cell line SHG-44 with adriamycin resistance.

**Figure 10:**
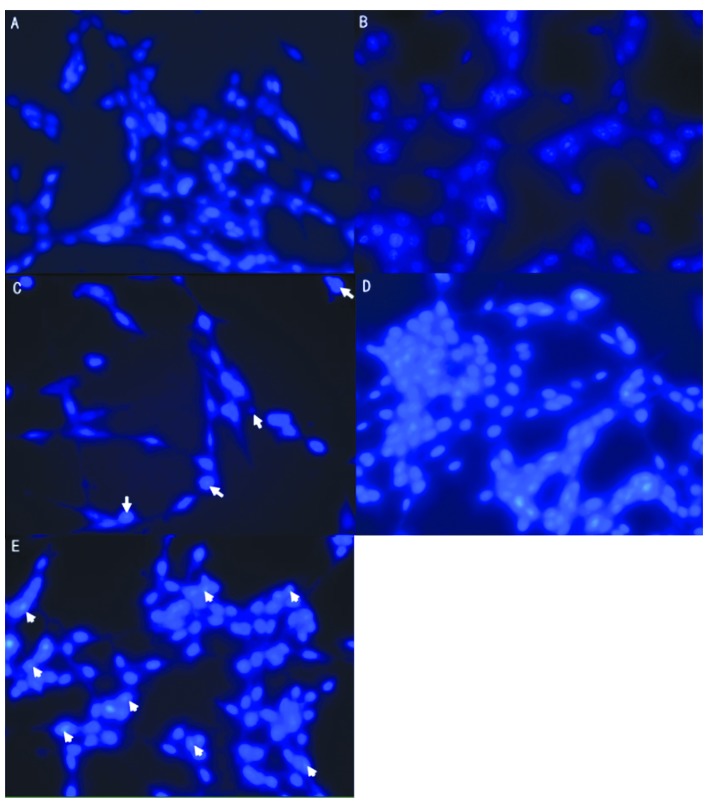
Apoptosis of SGH-44 and SGH-44/ADM determined by Hoechst 33258 staining and fluorescence microscopy (magnification, ×100). (A) SHG-44 and (B) SHG-44/ADM cells without any treatment. (C) SHG-44 and (D) SHG-44/ADM cells treated with 10 μg/ml ADM for 48 h. (E) SHG-44/ADM cells treated with 10 μg/ml ADM and 100 μM cyclosporin A for 48 h. Cells undergoing apoptosis are indicated by arrows. ADM, adriamycin; SHG-44/ADM, human glioma cell line SHG-44 with adriamycin resistance.

The apoptotic rate of SGH-44 cells increased from 3.52 to 70.97% when treated with ADM (Fig. 9). SGH-44/ADM cells were more resistant to ADM than SGH-44 cells, as the apoptotic rate showed a smaller increase from 3.19 to 15.89% when treated with ADM. However, the apoptotic rate of SGH-44/ADM cells was increased to 55.27% when treated with CsA, a MDR1 inhibitor. The apoptotic rate, determined by fluorescence microscopy, was accordant with that determined by flow cytometry (Fig. 10).

## Discussion

In the present study, a stable MDR glioma cell line, SGH-44/ADM, was generated by impulse ADM treatment. The effect of cryopreservation, recovery and withdrawal on SGH-44/ADM MDR cells was poor. SGH-44/ADM cells showed minimal differences as compared with parental cells, except that SGH-44/ADM cells were bigger in size, and with a wizened nucleus. However, SGH-44/ADM cells exhibited a slower growth rate and stronger excretion ability. As compared with SGH-44 cells, an increased number of SGH-44/ADM cells remained in G1 and S phase, as measured by flow cytometry. The MDR ability was associated with the upregulation of MDR1, PKCα and COX-2, but the expression of these genes was not associated with DNA methylation.

The cell model of MDR glioma has been described previously. Denecke *et al* (35) established a subline of the rat glioma cell line C6, named C6, 5×10(−7) Dox, by exposure to increasing doses of doxorubicin over five months. In addition to the typical cross-resistance to doxorubicin, daunorubicin, vincristine and etoposide, it was observed that a significant resistance of the C6, 5×10(−7) Dox cell line to irradiation was developed, which cannot be explained by Pgp expression. As well as the C6, 5×10(−7) Dox cell line, several other stable MDR human glioma cell lines had been previously established (36–38). All these previous studies failed to measure the resistance to ADM. Kang and Kang (38) established a dissociated cell system of human glioblastoma multiforme (GBM) cells, A172. GBM2 cells were established with resistance to 1,3-bis(2-chloroethyl)-1-nitrosourea and expressed CD133, CD117, CD90, CD71 and CD45 cell-surface markers.

In the present study, Pgp was shown to be upregulated in the MDR cell line SGH-44/ADM, which was concordant to a previous study (35). COX-2 is an inducible isoform enzyme that functions to generate prostaglandins from arachidonic acid. It has been previously observed that enforced expression of COX-2 results in enhancement in MDR1 expression and functional activity, which suggests the existence of a causal link between COX-2 activity and MDR1 expression (39). The protein kinase C (PKC) family functions in regulating cell proliferation and death, and is a key protein in the signaling pathway linking epidermal growth factor receptor to mammalian target of rapamycin (40,41). It had been observed that ribozymal inhibition of PKCα may trigger apoptosis in glioma cells (42). In the present study, PKCα and COX-2 were upregulated in SGH-44/ADM cells, which is consistent with previous studies (39,42).

Methylation of *MDR1*, *PKC*α and *COX-2* was not observed in the present study. The most significantly enriched pathway identified by MeDIP-Chip was SNARE interactions in vesicular transport (P=0.005). Three genes were involved in this pathway, including *SNAP47*, *VAMP4* and *VAMP3*. The other significant pathways were sphingolipid metabolism, endocytosis, GPI-anchor biosynthesis, collecting duct acid secretion, pentose phosphate pathway and glycine, serine and threonine metabolism. Enriched genes associated with endocytosis were *CHMP1B*, *ARRB2*, *PARD6B*, *TGFB1*, *VPS4B* and *CBLB*.

The human *VPS4B* gene is a homolog of yeast *VPS4*, and shares a high degree of similarity with mouse suppressor of K^+^ transport defect 1, which is associated with transmembrane transport (43). VAMPs, NSF, SNAP and Annexins are the main components of the protein complex involved in the docking and fusion of synaptic vesicles with the presynaptic membrane (44), and may function in trans-Golgi network-to-endosome transport (45). CHMP1B is a peripherally-associated component of endosomal sorting. CHMP1B is required for transport complex III (ESCRT-III), which is involved in the formation and sorting of endosomal cargo proteins into multivesicular bodies (46). VPS4B, VAMPs and CHMPs are components of the ESCRT-III (46), which suggests that the major mechanism of MDR in glioma is exocytosis.

In conclusion, SGH-44/ADM cells were shown to have minimal differences from parental cells, except for a larger cell size and a wizened nucleus. A larger proportion of SGH-44/ADM cells remained in G1 and S phase, as compared with SGH-44 cells. The MDR ability of SGH-44/ADM cells was associated with the upregulation of MDR1, PKCα and COX-2. The expression of these genes was not associated with DNA methylation, which suggested that the mechanism of MDR in SGH-44/ADM is by active efflux rather than DNA methylation. In addition, certain components of immune-associated reactions, including positive regulation of cytotoxic T-cell differentiation, were significantly enriched. These findings may provide a novel insight into the MDR of human gliomas.

## Figures and Tables

**Table I:** Specific primers for quantitative polymerase chain reaction.

Genes	Primer sequence
*PRPS1*	F: 5′-ATCTTCTCCGGTCCTGCTATT-3′ R: 5′-TGGTGACTACTACTGCCTCAAA-3′
*ARRB2*	F: 5′-TCCATGCTCCGTCACACTG-3′ R: 5′-ACAGAAGGCTCGAATCTCAAAG-3′
*GBA*	F: 5′-CATCCGCACCTACACCTATGC-3′ R: 5′-TGAGCTTGGTATCTTCCTCTGG-3′
*GAPDH*	F: 5′-GGAGCGAGATCCCTCCAAAAT-3′ R: 5′-GGCTGTTGTCATACTTCTCATGG-3′

F, Forward; R, Reverse; PRPS1, phosphoribosyl pyrophosphate synthetase 1; ARRB2, arrestin β2; GBA, glucocerebrosidase.

**Table II:** Resistance of SGH-44 and SGH-44/ADM to different drugs.

	IC_50_ (μg/ml)		
		
Drugs	SGH-44	SGH-44/ADM	RI
ADM	1.67±0.05	17.83±0.52	10.7
EPI	0.87±0.06	7.22±0.14	8.3
VCR	2.95±0.09	21.42±0.67	7.2
MMC	8.74±0.33	47.73±1.12	5.4
Ara-C	8.38±0.42	10.27±0.39	1.2
DDP	0.78±0.07	2.46±0.11	3.1

IC_50_, half inhibitory concentration; RI, resistance index; ADM, adriamycin; EPI, epinephrine; VCR, vincristine; MMC, mitomycin C; Ara-C, arabinfuranosyl cytidine; DDP, cisplatin; SGH-44/ADM, SHG-44 cell line with ADM resistance.
